# Yellow Fever Inoculation

**Published:** 1886-11

**Authors:** 


### YELLOW FEVER INOCULATION.

In a letter received by Dr. J. McF. Gaston, of this city, from Dr. Domingos Freire, of Rio de Janerio, dated August 26th, 1886, the following passages are of special interest to all who have kept up with the discussion of the claims of this prophylactic measure upon our Southern people:

"It is to be expected that the American Medical Association, whose influence is brought to bear upon Congress, will in the end succeed in obtaining the appointment of the commission, with which result I will be much gratified.

"The Brazilian government has now given me the means to establish, upon a proper basis, a laboratory of micro-biology, so that we can make all the investigations and demonstrations in a correct and becoming manner.

"I will forward to you shortly the statistics of the last epidemic, which are nearly ready for publication, as likewise a 'notice of the regeneration of the virulence of cultures of the attenuated microbe of yellow fever.' This last undertaking allows me to graduate the cultures from a fatal virulence to a vaccine attenuation. With these modified cultures, I can make the demonstrations upon animals at any time in any season of the year.

"It is expected that in a short time experiments with these cultures will be made in the laboratory of Brown Sequard, at Paris, and I will perhaps go there to witness them.

"I am deeply impressed with the reception extended to my labors by a large portion of the medical profession of the United States, and will be under obligations to you to do me the kindness of manifesting publicly my gratitude."

Dr. Gaston promises a translation of the concise treatise above referred to for our next number ; and this, in connection with the elaborate paper of Dr. Finlay, in the October number of the *American Journal of Medical Sciences*, will enable those who are open to conviction to understand the merits of yellow fever inoculation.

---

### ITEMS.

DR. N. O. HARRIS has been elected President of the Atlanta Society of Medicine.

ALL operations in the Atlanta Medical College are now made under strict antiseptic precautions.

DR. HUNTER P. COOPER was recently elected Professor of Chemistry in the Atlanta Medical College.

DR. STEPHEN NEAL, of Talbotton, recently visited Atlanta and gave THE JOURNAL a pleasant call.

WE return thanks to Messrs. Reed & Carnrick, of New York, for a copy of their new "diet tables," which we find very convenient and useful.

DR. WM. F. HOLT and Dr. K. P. Moore, two of Macon's most prominent physicians, are now in New York, where they will remain for several months.

DR. D. H. HOWELL, of this city, for a number of years assistant to the chair of surgery in the Atlanta Medical College, is in New York attending the polyclinic.

THE clinics at the Atlanta Medical College are better this session than we have ever known before. The surgical and eye and ear clinics are especially interesting.

Dr. J. D. WILSON has resigned the professorship of chemistry in the Atlanta Medical College and has removed to New York, where he is engaged in the practice of medicine.

THE OPENING OF OUR MEDICAL COLLEGES.—The medical colleges of Atlanta opened their regular winter sessions the first week in October. The introductory lecture at the Southern was delivered by Professor Roy. There was a good class present. Dr. Hunter P. Cooper, the newly elected Professor of Chemistry in the Atlanta Medical College, delivered the opening address at this college to one of the largest classes ever assembled in the college.

INGLUVIN.—A very learned name for a remedy is ingluvin. It is the essential principle of the gizzard, and bears the same relation to poultry that pepsin does to the higher animals. The honor of its discovery and utilization, in its crude state, remotely dates with the Chinese gastronomer, as well as to the Caucasian chemist in its refined condition. From time immemorial the inhabitants of the Celestial Empire have used the gizzard of chickens and ducks in nearly all made dishes. Their writers have recommended the practice as a sovereign treatment of dyspepsia, weak stomach and vomiting. A favorite prescription of Chinese physicians for chronic indigestion is to cut up and digest chicken gizzards in hot water until they are reduced to a pulp, and then add some spices. A tablespoonful or two of the resulting paste is taken at each meal until the patient has entirely recovered. From China the practice passed to other parts of Asia, and was adopted here and there among the Mediterranean people. Strange to say it was never learned by the great nations of Europe until the latter part of the present century. On the other hand, the organic chemists of Europe discovered about 1850 a powerful nitrogenous radical in the gizzard. Experiments thereafter showed it to possess many of the qualities of pepsin. These experiments led to its isolation. Numberless experiments have proven it to be a very valuable addition to therapeutics. Where pepsin refuses to act, and where, in severe cases, it has even been rejected by the stomach, ingluvin effected relief rapidly and with the greatest ease.

Ingluvin has the remarkable property of arresting certain kinds of vomiting—notably the vomiting of pregnancy. It is a stomachic tonic, and relieves indigestion, flatulence and dyspepsia. Ingluvin is prepared by the far-seeing chemists, Wm. R. Warner & Co., of Philadelphia. It is made from selected gizzards, and is so carefully extracted as to be free from all foreign organic bodies. It is already known and appreciated by the medical profession.—*American Analyst, August, 1886.*

CASTRATION IN MENTAL AND NERVOUS DISEASES.—The October number of *The American Journal of the Medical Sciences* contains a symposium by the leading authorities on this subject in the United States, Great Britain and on the Continent. Sir Spencer Wells maintains that the operation of oöphorectomy, or the removal of normal ovaries, is one which may be advised in some cases of uterine fibroids, and in uncontrollable uterine hemorrhages. That it is to be resorted to in certain malformations of the genital organs, deformities of the pelvis, and accidental obstruction of the vagina. That the right to use it is very limited in cases of ovarian dysmenorrhœa or neuralgia, and only when they have resisted all treatment, and life or reason is endangered. That in nearly all cases of nervous excitement and madness it is inadmissible. That it should never be done without the consent of a sane patient, to whom its consequences have been explained. That the excision of morbid ovaries and appendages should be distinguished from oöphorectomy, and ought not to be done without the authority of consultation, as in most other cases of abdominal section. That in nymphomania and mental diseases it is, to say the least, unjustifiable.

Prof. Hegar holds that castration is indicated in a psychosis evoked or maintained by pathological alteration of the sexual organs, and in a neurosis originating from the same source, as soon as this imperils life or hinders all occupation and all enjoyment of life. The indication is also present when that disease represents only one causal factor in the genesis of the affection, without the removal of which a cure is not to be thought of. The remaining causes of suffering must be in this case accessible to treatment. Other milder methods of treatment must have been tried previously without success, or, as in the case of many small tumors of the ovaries and tubes, must from the outset give no promise of success. Castration must actually affect the cause which occasions or keeps up nervous irritation. The operation will thus be of use when a degenerated or dislocated ovary represents the irritative focus, or as soon as a greatly swollen and retroflexed uterus presses on the sexual plexus and the organ is brought into a state of atrophy. Castration promises success when the bleeding and anæmia occasioned by a fibroma play an important part in the maintenance of a psychosis, so that a cure does not appear possible without getting rid of that evil; but castration is absolutely no universal remedy for any neurosis originating from a genital-organ disorder, or kept up by the same. The cessation of ovulation will avail nothing if the irritation starts from the nerves which are compressed in a shrunken cicatrix of the broad ligament, or elsewhere in a cicatrix of the pelvic connective tissue.

Dr. Battey writes that he has performed castration for the relief of mental and nervous disorders, which may be divided into three classes: oöphoro-mania, oöphoro-epilepsy, oöphoralgia. He uses the terms oöphoro-mania and oöphoro-epilepsy instead of hystero-mania and hystero-epilepsy, because clinical experience teaches him that these disorders are dependent upon a nervous irritation proceeding from the ovaries and not from the uterus. He finds the disorders existing (*a*) in cases in which he recognizes organic diseases of the ovaries, and is not able to recognize any organic disease of the uterus; (*b*) in cases of uterine as well as ovarian disease, when the diseased ovaries are removed, the nervous disturbance disappears, notwithstanding the fact that a displaced or diseased uterus may remain. In his experience the time required for the disappearance of nervous disorders, after removal of the ovaries, has been quite variable. In general, epileptiform manifestations have ceased at once. Some of the cases have required for a time the tranquillizing effects of the bromides to ward off threatening symptoms, whilst others have needed nothing. His cases of mania have all been quite chronic, and the improvement has been slow. In oöphoralgia, in a few instances, the cure has been immediate and permanent. In the majority it has been slow and gradual, and in others nothing has been gained for even two years after the operation. In a few the long-established opium habit has proved a complete bar to recovery.

In his cases, which have had two years or more to test them, seven have been cases of oöphoro-mania; of them one was cured and four improved; nine were cases of oöphoro-epilepsy, all cured; twenty were cases of oöphoralgia and thirteen were cured and three improved.

THE LOCAL TREATMENT OF PSEUDO-MEMBRANOUS CROUP—INTUBATION OF THE LARYNX.—Dr. J. Lewis Smith, in an article in the October number of *The American Journal of the Medical Sciences*, expresses his belief that intubation is destined to be employed more generally than tracheotomy in the treatment of pseudo-membranous croup. He maintains that in all cases in which the obstruction is limited to the larynx and trachea, intubation relieves the dyspnœa as quickly, effectually and permanently as does tracheotomy. It gives, in most instances, complete relief for a time. If the respiration subsequently become embarrassed, and no benefit occur from cleaning the tube, tracheotomy may be required. Intubation may properly precede tracheotomy in most cases.

Not a few parents, in the middle and lower classes, allow their children to die rather than consent to this operation. On the other hand, few parents will object to intubation, and when they see the relief which it produces they will probably consent more readily to tracheotomy if the dyspnœa should return. If only one of these operations be performed, statistics thus far show nearly as good a result from intubation as from tracheotomy.

Now that diphtheria has become so common, the physician should be provided with the necessary instruments for intubation whenever diphtheria appears in his locality. Alkaline and trypsin inhalations, properly and almost constantly used, and intubation performed early, when the patient begins to suffer from dyspnœa, would probably prevent the necessity of tracheotomy in a large proportion of instances. But if such treatment do not fully relieve the dyspnœa, it will, in most instances, so diminish it and retard the progress of croup that the physician, remote from help and unfavorably situated for the performance of tracheotomy, will have ample time to prepare for this operation. Intubation may prevent the need of tracheotomy, but if not it presents no hindrance to it.

SOME people in Georgia think it is all right to dissect subjects "from the North," but it is just simply awful to dissect a Georgian. Wonder what they would think if they knew how many Georgians were dissected in "the North" every winter? The South has always furnished more subjects for the dissecting rooms of the North and West than was ever furnished by them to the South. Seriously we doubt whether there was ever a subject sent from any of the Northern States to a dissecting room in Georgia, and it is about time the Legislature knew this fact.

THE COST OF INFANT FOODS.—The manufacturers of lactated food furnish us with the following: "One of the greatest objections that has been made to the use of the various prepared infant foods upon the market has been their high cost. As it will be a matter of interest to the entire profession to know the comparative costs of the various foods, a careful computation of the cost of each class has been made, prepared according to the directions given for infants. The so-called milk foods or powders are found to be the highest, averaging to cost, when prepared ready for use, about nine cents per pint; next in cost is a class called Liebig's food, which average six cents or more a pint; next in a class of farinaceous foods, which cost nearly as much as the Liebig food. Below all these is lactated food, which costs but four cents per pint, making it the most economical food the profession can use. A dollar package of lactated food will give an infant one hundred and fifty meals, or sufficient to last about four weeks."

SUBSTITUTION.—Messrs. Battle & Co., the manufacturers of bromidia, have received the following letter from Dr. Geo. Springer, of Van Buren, Ohio : Gentlemen—In the case of "insomnia," which I reported to you in May last, and wherein it required seven drachm doses (hourly, 1 drachm) to produce sleep by bromidia bought at pharmacy in Findlay, it required but one drachm, repeated in one hour, to produce a good night's rest, of the sample bottle you sent me. I also use the bromidia (Battle & Co.) with the best results in "cholera infantum," and in "hysteria." I am satisfied that the article bought at Findlay was "spurious."

CONGENITAL HEREDITARY ATONIC DYSPEPSIA.—During a practice of twenty years, I have prescribed lactopeptine to patients of all ages, and have never been disappointed in its action when indicated. But I desire to speak in particular of its action in a case of congenital hereditary atonic dyspepsia in an infant to whom I began to administer this remedy on the third day after birth. Mrs. H. L. S., Langside, Miss., was delivered of a male child in whom there was manifested well-marked symptoms of atonic dyspepsia. The mother had been a victim of dyspepsia from girlhood and had inherited the malady from her mother.

The infant was put to the breast a few hours after birth and nursed readily, but almost immediately rejected the milk. Repeated trials all resulted in vomiting, followed by exhaustion. Other articles of food were tried, including cow's milk, etc., without improvement. The child was in great danger of starvation. On the third day, I began the administration of lactopeptine. The effect was immediate and almost miraculous. I ordered one-sixteenth of the adult dose to be dissolved in about two ounces of breast milk (drawn from a robust, healthy wet-nurse) and administered every two and a half hours. There was no more rejection of milk, except the usual vomiting of curdled milk, to relieve the crowded state of the stomach, which occurred occasionally, after the first ten days. Condensed milk, cow's milk (properly diluted and sweetened), Mellin's food, boiled bread (pap) were, after a while, substituted for breast milk, but always with lactopeptine. A steady improvement was manifest from the beginning, and kept up during the first dentition, which process was gone through with in a most satisfactory manner. No untoward diarrhœa or intestinal disturbance characterized this period, and, at ten months, the child was virtually cured of its dyspepsia, and could eat and digest ordinary food such as children of that age may do in good health. The parents of the child believe firmly (as I do) that lactopeptine saved their infant.

In cholera infantum, in diarrhœa, and in all of the disturbances of the alimentary canal, during dentition and early infant life, I find lactopeptine an ever-effective and reliable remedy. In adult dyspepsia, all are now familiar with its beneficial effects; but I should be glad if the profession would be induced to try it in the vomitings, diarrhœas and dyspepsias of infancy. I recall several babies whose lives I believe I could have saved had I known ten years ago what I do now of the ready adaptability of lactopeptine to infant ailments.—R. Walker Beers, M. D., Angola, La., in the *Medical Brief*.

PEACOCK'S BROMIDES.—Of this preparation Fred. B. Wood, M. D., 456 Broadway, Milwaukee, Wis., writes: "I have given Peacock's bromides a *thorough test*, and am pleased to state that after an experience of twenty-five years I have never found any remedy which acts so surely as this preparation does. I am sure that in the near future, especially in the treatment of the brain and nerves, it is destined to take the place of the older preparations to the benefit of both physician and patient."

THE ADMINISTRATION OF COD-LIVER OIL.—Dr. W. Washburn, of New York city, writes that he has long been in the habit of administering cod-liver oil in milk to both infants and adults. Milk is taken in the mouth and held there, and the spoon is first dipped in milk and then the oil is poured into it. Just as the oil is taken into the mouth the milk should be swallowed, and then another sip of milk taken. Children, if interrupted in nursing, readily swallow a teaspoonful of oil and then proceed with nursing as if nothing had happened. The oily nature of the milk seems completely to shield the mucous membrane of the mouth and throat from contact with the cod-liver oil.—*N. Y. Medical Record.*

METEOROLOGICAL SUMMARY FOR SEPTEMBER, 1886, STATION, ATLANTA, GA.

| | |
|---|---|
| Mean Temperature, | 73.0 |
| Highest Temperature, September 11th | 90.0 |
| Lowest Temperature, September 30th, | 53.0 |
| Monthly Range of Temperature, | 37.0 |
| Greatest Daily Range of Temperature, | 23.0 |
| Least Daily Range of Temperature, | 7.0 |
| Mean Daily Range of Temperature, | 18 0 |
| Prevailing Direction of wind, (7 a. m., 3 and 11 p. m.) | E. |
| Total movement of wind, | 5,983 miles. |
| Highest Velocity of wind, Direction and Date, 22 miles N.W., 12th. | |
| Total Precipitation. | .53 inches. |
| Number of Cloudy Days | 2.0 |
| " " Clear " | 14.0 |
| " " Fair " | 14.0 |

